# Designing Thiadiazoloquinoxaline-Based Conjugated Polymers for Efficient Organic Photovoltaics: A DFT/TDDFT Study

**DOI:** 10.3390/molecules29071580

**Published:** 2024-04-01

**Authors:** Taylor A. Dorlus, Juganta K. Roy, Jerzy Leszczynski

**Affiliations:** 1 Interdisciplinary Center for Nanotoxicity, Jackson State University, Jackson, MS 39217, USA; taylor.dorlus@icnanotox.org (T.A.D.); jroy@wtamu.edu (J.K.R.); 2 Clean Energy Materials Modeling Laboratory, Department of Chemistry and Physics, West Texas A&M University, Canyon, TX 79016, USA

**Keywords:** conjugated polymers, organic semiconductor, thiadiazoloquinoxaline, organic solar cells

## Abstract

Clean and renewable energy development is becoming frontier research for future energy resources, as renewable energy offers sustainable and environmentally friendly alternatives to non-renewable sources such as fossil fuels. Among various renewable energy sources, tremendous progress has been made in converting solar energy to electric energy by developing efficient organic photovoltaics. Organic photovoltaic materials comprising conjugated polymers (CP) with narrow optical energy gaps are promising candidates for developing sustainable sources due to their potentially lower manufacturing costs. Organic semiconductor materials with a high electron affinity are required for many optoelectronic applications. We have designed a series of organic semiconductors comprised of cyclopentadithiophene as a donor and thiadiazoloquinoxaline (TQ) as an acceptor, varying the π-conjugation and TQ-derivatives. We have employed density functional theory (DFT) and time-dependent DFT (TDDFT) to evaluate the designed CP’s optoelectronic properties, such as optical energy gap, dipole moment, and absorption spectra. Our DFT/TDDFT result shows that the energy gap of CPs is lowered and redshifted in the absorption spectra if there is no insertion of conjugation units such as thiophene and selenophene between donor and acceptor. In addition, selenophene shows relatively better redshift behavior compared to thiophene. Our work also provides rational insight into designing donor/acceptor-based CPs for organic solar cells.

## 1. Introduction

Using fossil fuels to drive the world’s economy impacts our earth by destroying the environment [1,2,3]. Moreover, increasing energy consumption and the rising cost of energy are the driving forces behind the development of new technologies to harvest solar energy. Organic solar cell (OSC) technology is a promising candidate for solar energy conversion compared to its inorganic counterparts due to its low cost, light weight, and potential use in flexible devices [4,5,6,7]. A typical OSC consists of a transparent conducting electrode (typically indium tin oxide), a metal top electrode, cathode and anode interlayers, and photoactive layers with the donor/acceptor blend or donor/accepter-based polymers. The organic photoactive layer acts as an organic semiconductor. The semiconductor layer generates electricity by harnessing solar photons based on the semiconductor bandgap and the wavelengths of light [5,8,9,10].

The last few decades have witnessed the rise of narrow bandgap organic semiconductors based on small molecules and conjugated polymeric materials [11,12,13,14,15]. Conjugated polymers (CP) are broadly utilized in OSC due to their customizable building blocks. However, this tunability presents challenges in optimizing the dissociation of electron-hole pairs, separating those pairs into free charges, and transporting the free charges to the electrodes simultaneously [16,17]. To avoid recombination, the high hole mobility of a polymer must be one of its optoelectronic characteristics. As a solution, conjugation facilitates better charge carrier capabilities. CPs generally consist of a π-conjugated sp2 carbon-based backbone surrounded by aliphatic side chains, responsible for, to a first approximation, the optoelectronic properties and solution processability, respectively. The former remains the focus for much of the research in this area, primarily due to the inferiority of these materials relative to inorganic semiconductors on many optoelectronic performance metrics [18]. Organic conjugated material can be converted into electron-rich or electron-donating compounds by incorporating donor–acceptor units. Donor–acceptor-based π-conjugated polymers have theoretically proved to be highly efficient in this substitution based on having a large absorption coefficient, low band gap and environmental stability. Moreover, proper side chain and backbone engineering contribute to an ideal donor–acceptor unit [19].

A salient design feature is the cross-conjugated donor, which allows intricate control of structure–property relationships, raises the highest occupied molecular orbital (HOMO), affords adjustments to molecular indices such as bond length alternation (BLA), and promotes a highly planar conjugated backbone [20,21]. The strong, pro-quinoidal thiadiazoloquinoxaline (TQ) acceptor is critical for lowering the lowest unoccupied molecular orbital (LUMO) and promoting strong electron correlations to form and stabilize unpaired spins in the long-chain limit [13,15]. In addition, Boudreault’s study revealed that donor–acceptor-based polymers maximize light absorption in the active layer of the polymer-based solar cell [22]. Therefore, conjugation within donor–acceptor polymers makes the cell more flexible and susceptible to reactions to achieve the desired results [23]. A CP adopting a donor–acceptor (D–A) type alternating structure represents a promising group of organic semiconductor materials for electronics. Careful consideration of positional isomerization of D-A polymers and the number of fused thiophene rings in the polymer backbone are effective strategies for designing polymers with improved photophysical parameters for OSC technology, as these factors impact intramolecular conjugation and intrinsic charge mobility [24,25]. Notably, OSCs have attracted considerable attention from researchers owing to their inherent advantages, such as flexibility, lightweight, solution-processability, and potentially low-cost fabrication [26].

Benzobisthiadiazole (BBT) is a well-known acceptor that is commonly used, in addition to benzothiadiazole (BT). The use of the derived TQ acceptor in OSC is very promising for increasing the photo-conversion efficiency, owing to its electron-deficient nature and good planarity. As an acceptor in D-A type semiconductors, TQ acts as a p-type semiconductor [27]. Moreover, some of the advantages of this acceptor compared to the slightly stronger BBT acceptor are that the TQ acceptor can be functionalized with various aryl or alkyl groups, thus improving the solubility immensely [15]. Previous TQ-based polymers have proven helpful as active materials in OSCs and OFETs [14,28,29]. The Azoulay group recently reported high-spin OSCs with long-range π-electron correlations, interrelated (opto)electronic functionalities, and robust stability. The Azoulay group synthesized poly(4-(4-(3,5-didodecylbenzylidene)-4*H*-cyclopenta [2,1-b:3,4-b′]dithiophen-2-yl)-6,7-dimethyl-[1,2,5]-thiadiazolo [3,4-g]quinoxaline), a DA CP that overcomes conjugation saturation behavior, exhibits a very narrow bandgap (Eg < 0.6 eV), and is a ground-state triplet [13]. However, the TQ acceptor building blocks, which enabled a narrow bandgap CP, need to be improved substantially [30]. The limited structural attributes and electron-withdrawing strength of the TQ building block are design elements that can be significantly improved upon further investigation. The highly electron-deficient TQ acceptor can exhibit varying inductive effects based on the electron-donating groups (methyl, thiophene, selenium) at its terminal ends. Selenium enhances polarizability, which results in a redshift in absorption spectra [31,32]. Moreover, different studies [33,34] established that the longer π-conjugation in the DA polymers exhibits a narrow band gap and moves the absorption spectra to the higher wavelength, which is worthwhile in harvesting longer wavelength photons.

The choice of acceptors and π-conjugation in D/A-based organic semiconductors is significant in designing a narrow bandgap semiconductor. Computational modeling and prediction of the properties offer a cost-effective and fast screening of new CPs. Based on reference [13], we have modeled seven CPs using different TQ-derived acceptors with donor CPDT to investigate their impact on the HOMO-LUMO gap. We assessed the frontier molecular orbital energy and their gap, including the UV-Vis spectra with different functionals. Our study explored different optoelectronic properties of D-A-based semiconductors, varying π-conjugation and TQ-derivatives. We found that the π-conjugation in between D and A units reduces the HOMO-LUMO gap and induces redshifts of the UV-Vis absorption spectra.

## 2. Results and Discussion

**Figure 1:**
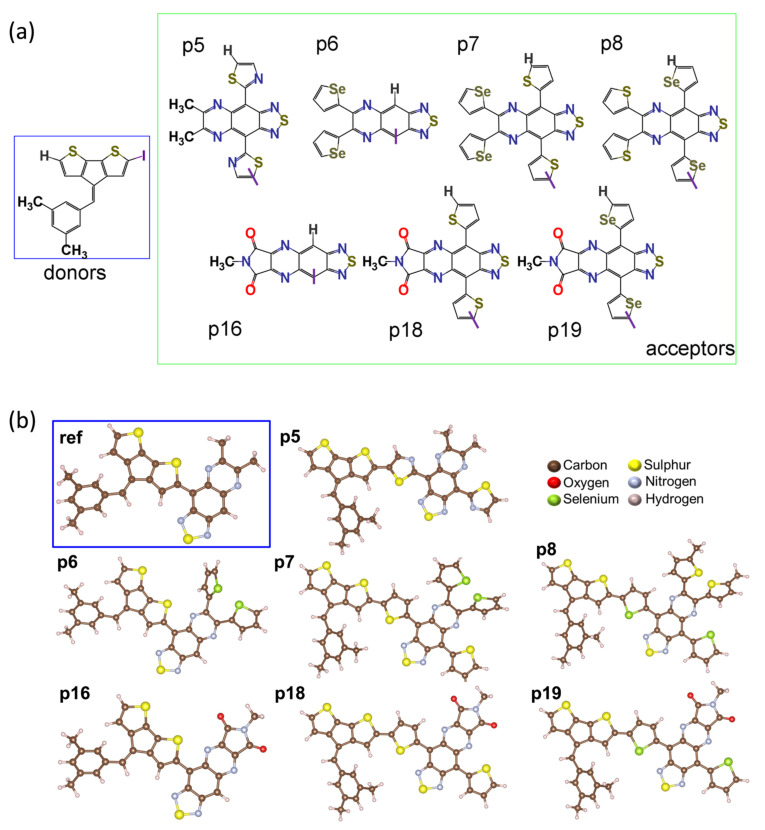
(**a**) Different acceptors with the donor used in this study. Here, the terminal ends or connecting ends are denoted by I, where blue and green box indicates the donor and the acceptors, respectively. (**b**) Optimized geometry of monomers of donor–acceptor-based conjugated polymer-based semiconductor at the level of theory DFT/B3LYP/6-311G(d,p). In the blue box in (**b**), the reference molecule is presented. VESTA software (v3.5.8) was used to visualize geometry.

We designed seven donor–acceptor-based CP semiconductors alternating cyclopentadithiophene (CPDT) and thiadiazoloquinoxaline (TQ) units. The structural and electronic characteristics of the monomers and oligomers are examined computationally, and the results obtained from the calculations are discussed in this section. We evaluated the optoelectronic properties of the π-conjugated monomers and oligomers, analyzing the molecular geometry, frontal molecular orbitals (FMOs), energy gap, and absorption spectra in the gas phase. It is well-known that the band gap of the conjugated systems is directly related to the molecular structure. Therefore, before explaining the HOMO-LUMO energy gaps and absorption spectra of the studied compounds, we discuss the calculation results associated with the structural parameters of monomers and oligomers. The optimized geometry of the designed CPs and acceptor fragments is listed in Figure 1. The improved solubility and processability of reported TQ-based acceptors due to favorable side chain positioning increases electron density and lowers the band gap to below 1 eV, making them suitable for use in organic photovoltaics [15]. The design of the polymers studied in Figure 1 is influenced by such experimental findings to produce efficient solar cell polymers. To design D-A-based monomers using a donor group, CPDT, and acceptor, TQ is used. Figure 1a shows the different TQ derivatives used in this study along with CPDT. We used selenophene (p6, p7) and thiophene (p8) for substituents in TQ instead of alkyl groups. The π-linkers thiazole (p5), thiophene (p7, p18), and selenophene (p8, p19) have been inserted in between D/A pairs to investigate the structure–property relationship. Furthermore, the TQ acceptor includes the electron-deficient five-membered ring N-methyl maleimide (p16, p18, and p19).

### 2.1. Frontier Molecular Orbitals and Energy Gap

**Figure 2:**
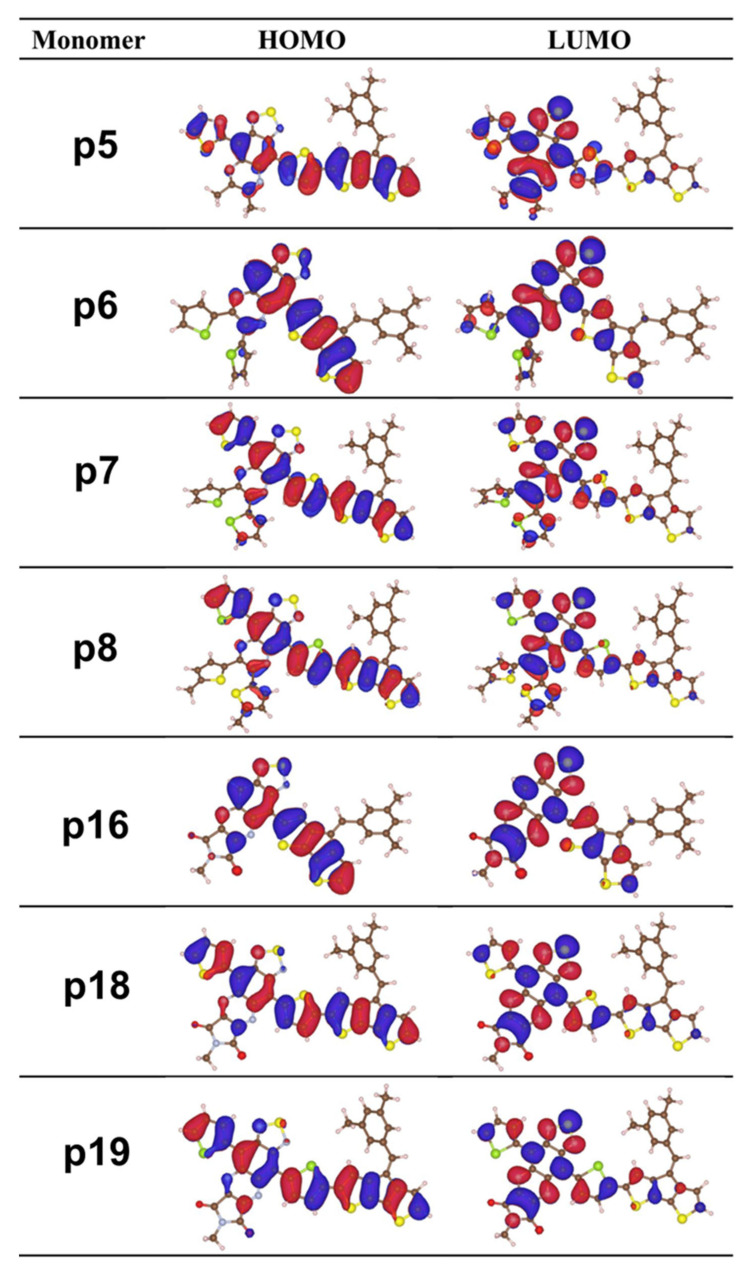
DFT-computed frontier molecular orbitals for all polymers at the B3LYP level of theory. Electron density distribution of HOMO and LUMO plotted using ISO = 0.02 au.

To obtain insight into the effect of TQ-derivative monomers and oligomers on the FMOs and their energy gap, the orbital energies were computed by the DFT/B3LYP/6-311G(d,p) level of theory in the gas phase. The visual representation of the electronic density distribution map of FMOs of the monomer subunit of the studied conjugated polymers at the B3LYP level of theory is presented in Figure 2. It is noted that both the HOMO and LUMO distribution shows π-molecular orbital characteristics. The contour plots of the molecular orbitals show that HOMO is mainly centered on the donor CPDT and extended up to the TQ acceptor via π-conjugation for all the studied monomers. However, LUMO is mainly located on the TQ-derivatives and, to a certain extent, on the π-conjugation unit and CPDT. This type of spreading of electronic cloud distribution confirms the strong intramolecular charge transfer (ICT) behavior [26,27,28]. In D-A conjugated polymers, the ICT process from the donor to acceptor moieties is dominated by the electron-donating/withdrawing ability of the donor/acceptor materials and their structure arrangement. The ICT is involved in the efficient charge transfer from donor to acceptor, and it can be evaluated by the overlapping nature of HOMO and LUMO orbitals over π-conjugation. Figure 2 indicates the favorable charge transfer induced by HOMO to LUMO, which will enhance the redshift of the designed CPs absorption spectra [35]. It is evident that the LUMOs of p6 and p16 are slightly extended to CPDT, as there is no π-conjugation unit. The density distribution map of the remaining dimer, trimer, tetramer and pentamer subunit oligomers is depicted in Figures S3–S6. From these plots, it is observed that the distribution of HOMO and LUMO is like monomers.

**Figure 3:**
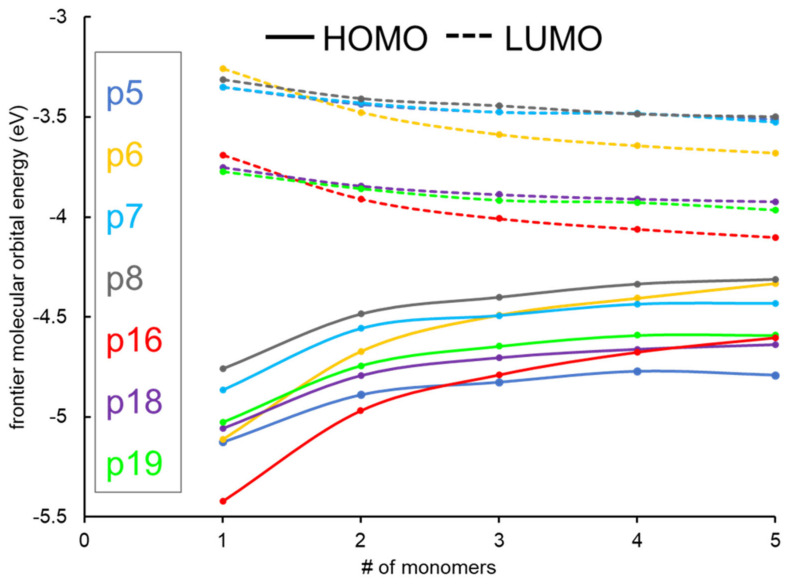
The distribution of HOMO and LUMO of different D-A polymers with the number of monomers up to 5 repeating units.

The computed $E_{HOMO}$, $E_{LUMO}$, $E_{gap}$, $E_{abs}$, $E_{b}$, ground state dipole moments ($\mu_{D}$), oscillator strengths (f), and major contributions for the H→L transition in the case of $S_{0}\to S_{1}$ transitions for the studied monomers and oligomers are tabulated in Table 1. The energy of HOMO and LUMO of the oligomers are plotted in Figure 3. The HOMO energies of the oligomers increase with the number of monomers, while the LUMO energies decrease irrespective of TQ derivatives (Table 1 and Figure 3). Consequently, $E_{gap}$ values of the oligomers decrease from monomer to pentamer. It is evident to mention that the percentage increment of HOMO energies is ~15% in the case of p6 and p16, while the other oligomers lie between 6.5 and 8.9% from monomer to pentamer. The main difference in the structure of p6 and p16 is acceptor TQ-derivatives directly attached to the CPDT; in the case of the other oligomers, there is a π-conjugation moiety. The diminished H→L contributions as monomer subunits increase is due to these different structural attributes.

**Figure 4:**
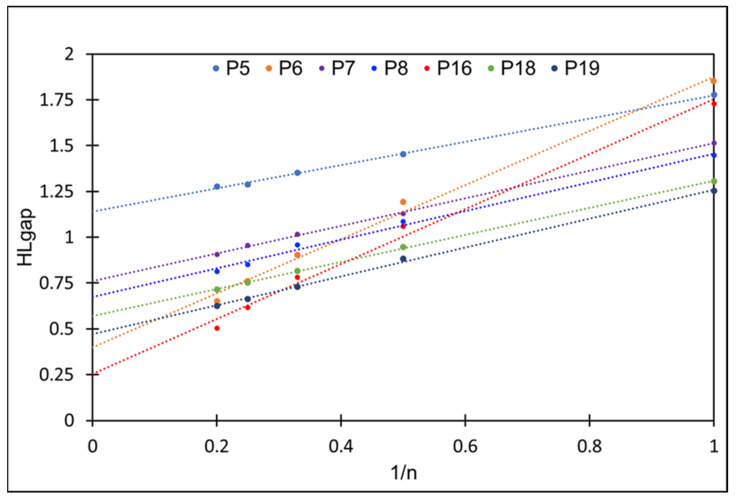
HOMO–LUMO energy gap ($E_{gap}$) as a function of the reciprocal of oligomer length (1/*n*) where *n* is the number of monomer units in a conjugated polymer. Level of theory DFT/CAM-B3LYP/6-311G(d,p). Dotted lines are fitted curves.

The $E_{gap}$ of polymers can be predicted by the linear extrapolation of $E_{gap}$ vs. 1/*n* to the polymer limit (n → ∞) using the computed $E_{gap}$ values of monomers and oligomers, as depicted in Figure 4. The extrapolated $E_{gap}$ of polymers is in the range of 0.25–1.15 eV, and these values are within the band gap of the semiconductor. The extrapolation of the $E_{gap}$ of the polymer is well established elsewhere [36] and depicted in Figure 4 for the studied CPs. Increasing the monomeric units from monomer to pentamer reduces the overall $E_{gap}$ as the HOMO energy increases and the LUMO energy decreases. Incorporating a five-membered unit between D and A decreases the slope of the fitted line, while the absence of a π-conjugation unit results in a steeper fitted line, yielding narrow $E_{gap}$ polymers.

Thiophene is a commonly used substituent that increases power conversion efficiency by well over 7% for photovoltaic devices. It has suitable HOMO energy levels, and thus, $E_{gap}$ enables a more π-extended conjugated backbone [37,38]. Despite the effectiveness of thiophene, selenophenes are the alternative to thiophenes, allowing direct substitution in conjugated systems. This heteroatom substitution strategy effectively tunes the properties of organic electronic materials without increasing their carbon content [8], which may adversely impact the cost and processability. Selenophene has a more pronounced quinoidal character that facilitates and enhances π-conjugation along the extended polymer chain, so the incorporation of selenophene positively affects charge mobility [38]. Replacing the thiophene unit with selenophene, either between D and A or as the substituents in the TQ acceptor, reduces the $E_{gap}$ (p5 vs. p6–p8 and p18 vs. p19), as shown in Table 1 and Figure 3. In contrast, the p6 and p16 oligomers exhibit lower $E_{gap}$ values on the extrapolated line of $E_{gap}$ vs. 1/*n* (Figure 4). The thiophene/selenophene unit makes the backbone zigzag, which impedes the charge transfer. In the case of p16–p19, modifying the TQ acceptor by adding N-methylmaleimide (thiaziazoloquinoxalineimide) reduces the $E_{gap}$ by lowering the LUMO values compared to TQ.

### 2.2. Dipole Moment

The difference in electronegativity between the electron-accepting and donating moieties with the organic π-conjugated polymer gives rise to a dipole moment. The ground state optimization of polymers in this study allows the measurement of a molecular dipole moment to be extracted from the DFT output. The Hilde research project concluded that polymer sidechains with dipole moments cannot be seen electronically as innocent spectators. The dipole moment has an effect that lowers the coulomb attraction between electrons and holes, thereby facilitating greater charge separation between the molecules [39]. A large dipole moment is a known contributor to more extended charge carrier capabilities, which implies that the lower dipole moment reduces the chance of effective charge generation.

Based on this correlation, in the case of the monomer, the B3LYP functional gives the favorable measurement of the charge separation constant for all polymers except for p5–p8. Referencing the third to last column in Table 1 that exhibits the calculated dipole moments under the specified functional, oligomers p5 through p8 have dipole moments well under 4.0 Debye for the monomer subunit. In contrast, p16, p18 and p19 display exponentially higher dipole moments at 7.1, 5.8 and 6.2 Debye, respectively. The length of conjugation strategically influences the increase in the dipole moment based on the ground state optimization in the case of some studied polymers.

Reduced electron-hole coupling can be caused by steric or electrostatic hindrance, which is more common as the conjugation length increases in the polymer. The dipole moments for the dimer subunits with the B3LYP method are significantly higher by ~2.00 Debye or more in the case of p7, p8, p16, and p19. Observed values of p6 and p18 reveal that the increase in subunit has almost little to no effect, with a difference value of 0.34 Debye and 0.05 Debye, respectively.

The molecular polarity parameter of the tetramer subunit significantly increases for p6 and p16 with difference values in the range of 3.0 to 3.4 Debye. This finding shows that the repeated increase in polymer subunits positively affects dipole moment, thus decreasing steric hindrance within the molecule for better optical absorption. As the conjugation of the organic polymers increases, the charge separation also increases. The side chains of the studied polymers also play a critical role in this material being used as a transporter for electric charge. A review of polymer applications in solar cells confirms that improving charge carrier mobility and reducing recombination within studied species are imperative strategies to enhance the device efficiency of OSC [2]. The studied oligomers are capable of this improved performance based on ground-state dipole moment results.

### 2.3. UV-Vis Analysis

**Figure 5:**
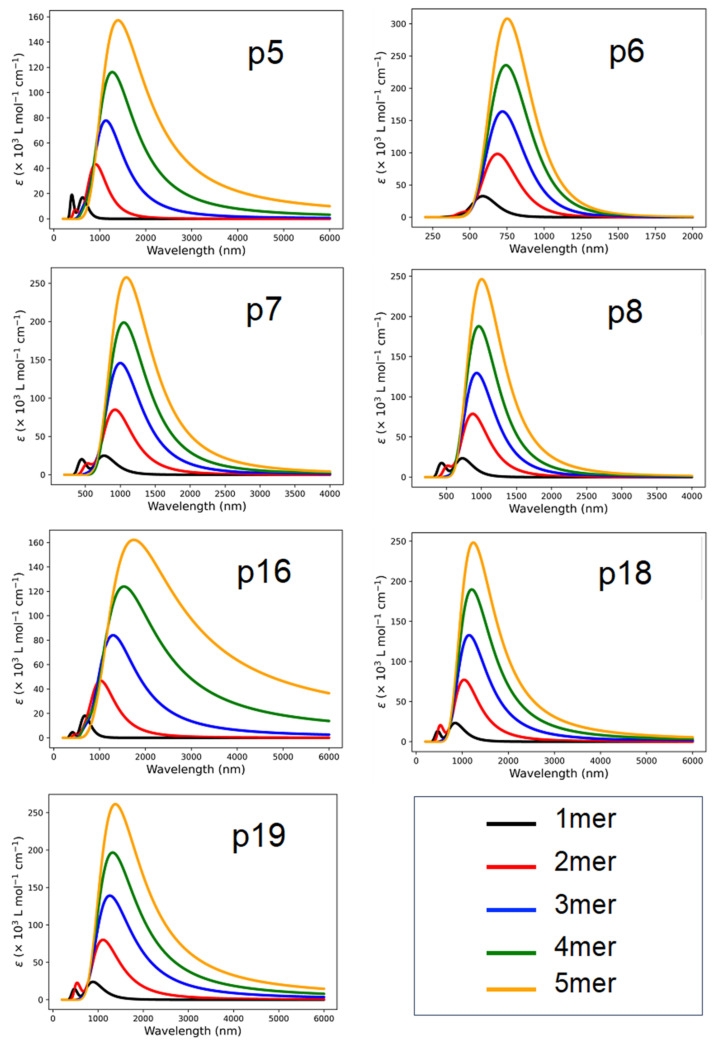
Computed UV-Vis absorption spectrum, molar absorption coefficient vs. wavelength, for donor–acceptor fused polymers at the CAM-B3LYP/6-31G(d,p) level of theory with the lowest 10 singlet excitation states.

The characteristic of light absorption of any CP is one of the factors to be considered during the design of polymer-based semiconductors to increase efficiency. Light absorption efficiency depends on the $E_{gap}$ of the CPs and can be tailored by appropriate D/A pairs, including the π-linkers. The excited energy, corresponding to the $E_{gap}$, of a molecule is the minimum energy to excite an electron from HOMO to LUMO for an electronic transition. After the electronic transition, it is necessary to separate the excitons (electron and hole pairs) in the donor for the intramolecular charge transfer. The exciton binding energy ($E_{b}$) can be estimated by the energy difference between the electronic and optical band gap ($E_{b}=E_{gap}-E_{abs}$), where $E_{abs}$ is the first singlet excitation energy. To assess the absorption properties of the oligomers (monomer to pentamer), we simulated the UV-vis spectra of all the designed CPs using TDDFT at the B3LYP/6-311G(d,p)//CAM-B3LYP/6-311G(d,p) level of theory in the gas phase. The computed UV-Vis absorption spectra of the oligomers are shown in Figure 5, and the $E_{abs}$, $E_{b}$, and oscillator strength (*f*) are listed in Table 1.

Figure 5 reveals that the UV-Vis spectra of all the designed CPs have an excellent response to the solar spectrum from monomer to pentamer. Responses from UV to near-infrared light (NIR) region, specifically for p6 and p16, extended absorption up to 1500 nm and 1800 nm, respectively. Furthermore, the UV-Vis spectra suggest that there are two absorption bands in existence: (1) smaller bands in the visible region and (2) a wide absorption band in the NIR region. The slight absorption peak between 300 and 700 nm can be ascribed to the heterocyclic units embedded in the structure of the polymers. Double absorption is a well-known characteristic of donor–acceptor-based copolymers [35]. A polythiophene-based DFT study suggests that absorption in this range is due to $\pi \to \pi^{*}$ transitions of aromatic substituents [40]. All the lowest energy electronic transitions and maximum absorption considered here mainly correspond to the transition from HOMO to LUMO. The ability of these oligomers to possess a $\lambda_{max}$ value in both the visible light and near-infrared regions allows them to be potential candidates for integration in optoelectronic and biomedical devices.

It is noted that when acceptor TQ-derivatives are directly coupled with donor group CPDT, there is a significant redshift (p6) in UV-vis absorption compared to its counterparts with π-linker (p7). Also, between the thiophene and selenophene π-linkers, the selenophene-infused oligomers (p8) shift the absorption bands towards the higher wavelength relative to the thiophene-infused oligomers (p7), as shown in Table 1 and Figure 5. The high absorption value shows the potential of good charge transport to facilitate electro-optical devices with a favorable efficiency. Advancements such as this one are challenging in the research of organic conjugated polymers because harvesting a low-bandgap with strong absorption in the second near-infrared (NIR-II) region is uncommon due to the scarcity of an effective molecular design strategy [41].

## 3. Computational Details

All calculations were performed using the Gaussian16 software package [42], with visualization performed using VESTA [43]. Ground state geometry optimizations were performed using a density functional theory (DFT) approach at the B3LYP/6-311G(d,p) level of theory. The basis set 6-311G(d,p) performed well enough to capture the frontier molecular orbital energies of the conjugated copolymers [44,45,46,47,48]. For the selenium (Se) atom, the effective core potential basis set, LANLDZ, was used [49]. Harmonic vibrational frequencies for the monomers were computed to confirm that all the stationary points on the potential energy surface were in minimum energy.

The B3LYP optimized ground state geometry results were combined with TDDFT theory calculations using the long-range corrected hybrid functional CAM-B3LYP, employing the same 6-311G(d,p) basis set, for singlet excitation. The vertical singlet excitation energies were estimated at their ground-state optimized geometries by considering the 10 lowest-lying singlet excited states in the gas phase to simulate the UV-vis absorption spectra within the conventional TDDFT formalism. To compute the HOMO and LUMO energies $E_{gap}=\left|E_{HOMO}-E_{LUMO}\right|$) the B3LYP functional was used for all monomers to oligomers (*n* = 1 to 5).

The B3LYP method employs a 20% Hartree–Fock (HF) exchange that provides preferred results for ground state properties, but it has some limitations when predicting charge transfer excitation energies [50]. This functional can provide a redshift in absorption spectra, but due to poor performance in describing excitation energies, it cannot directly predict the spectrum of absorption for donor/acceptor-based conjugated polymers. In contrast, the coulomb-attenuating method of B3LYP, known as CAM-B3LYP, is a long-range corrected functional that is used to calculate charge transfer excitation and is comprised of 65% long-range and 19% short-range HF exchange interactions [50,51]. According to Tsuneda et al., a long-range correction has solved the underestimations of charge transfer excitation energies and oscillator strengths in time-dependent Kohn–Sham calculations and has improved poor optical response properties such as hyperpolarizability in coupled-perturbed Kohn–Sham and finite-field calculations [52].

**Figure 6:**
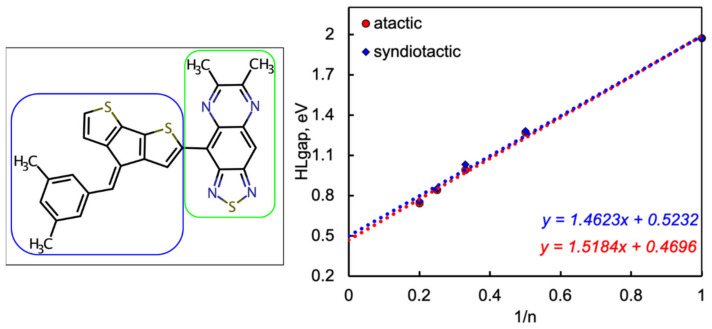
Reference D-A conjugated polymers (**left**) and benchmarking calculation of parent organic semiconductors showing the $E_{gap}$ vs. 1/*n*, where *n* indicates the number of repeating units (**right**). Blue and green box indicates the donor and the acceptor, respectively.

To validate our functional, we computed the $E_{gap}$ of the reference CP, and we plotted in Figure 6 the linear extrapolation of $E_{gap}$ as a function 1/*n* to the polymer limit (*n* → ∞), which provides 0.52 eV, consistent with experimental results and the reported DFT values in the literature [25]. We computed the atactic and syndiotactic conformer of the reference CP; however, the $E_{gap}$ does not deviate too much from the experiential values.

## 4. Conclusions

We designed novel D-A type polymers consisting of cyclopentadithiophene (CPDT) as a donor and thiadiazolquinoxaline (TQ) units as an acceptor based on the reference polymer to be used in organic solar cells. Different π-linkers such as thiazole, thiophene, and selenophene have been infused between CPDT and TQ to tune the optical properties. We conducted a systematic ground and excited state investigation on the optoelectronic properties of the designed polymers via DFT and TDDFT approaches. The computed HOMO-LUMO gaps of the designed oligomers have a range of 0.25–1.13 eV, whereas the $E_{gap}$ of the reference polymer is 0.5 eV. Our calculations revealed that the substitutions in the TQ acceptor using selenophene or N-methylmaleimide are conducive to making the acceptor more electron deficient, which leads to the lower energy gap between HOMO and LUMO in p6 and p16, respectively. Our results suggested that using a selenophene π-linker improves light-harvesting efficiency by enhancing backbone planarity and ICT behavior, outperforming other π-linkers. Simulated UV-Vis spectra for all the designed polymers are extended to the near-infra-red (NIR) region with one broad absorption band, except p5. Moreover, the exciton binding energy ($E_{b}$) is lowest in the p16 oligomer, leading to significant electron-hole pair separation after excitation. Considering the above factors, p6 and p16 would be preferable CPs for OSCs. Our work offers valuable guidance for designing D/A polymers with TQ acceptors for enhanced organic photovoltaic device performance.

## Figures and Tables

**Table 1:** Computed at the level of theory B3LYP/6-311G(d,p) for ground state and B3LYP/6-311G(d,p)//CAM-B3LYP/6-311G(d,p) for TDDFT calculations.

Name	*n*	*E_HOMO_* (eV)	*E_LUMO_* (eV)	*E_gap_* (eV)	$E_{abs}/\lambda_{max}$ (eV/nm)	*E_b_* (eV)	*µ_D_* Debye	*f*	$S_{0}\to S_{1}$ (Major Contributions)
**P5**	1	−5.13	−3.35	1.78	2.11/587.39	0.334	1.53	0.799	H→L (83.9%)
	2	−4.89	−3.44	1.45	1.79/691.15	0.341	2.30	2.293	H→L (71.6%)
	3	−4.82	−3.48	1.35	1.70/728.54	0.352	1.26	3.730	H→L (59.8%)
	4	−4.77	−3.48	1.29	1.64/755.73	0.353	3.80	5.063	H→L (48.2%)
	5	−4.80	−3.51	1.28	1.63/759.91	0.354	3.28	7.089	H→L (40.4%)
	∞			1.13					
**P6**	1	−5.11	−3.26	1.85	1.98/726.97	0.126	3.41	0.417	H→L (95.9%)
	2	−4.67	−3.48	1.19	1.35/917.63	0.157	3.75	1.043	H→L (90.2%)
	3	−4.49	−3.59	0.90	1.06/1142.61	0.183	6.37	1.887	H→L (84.1%)
	4	−4.41	−3.64	0.76	0.96/1292.19	0.197	7.66	2.754	H→L (78.0%)
	5	−4.33	−3.68	0.65	0.86/1439.09	0.209	10.52	3.590	H→L (71.5%)
	∞			0.40					
**P7**	1	−4.87	−3.35	1.52	1.70/729.69	0.184	0.56	0.577	H→L (91.8%)
	2	−4.56	−3.43	1.13	1.40/890.94	0.263	2.27	1.782	H→L (72.9%)
	3	−4.49	−3.48	1.01	1.29/954.14	0.283	4.39	2.788	H→L (60.0%)
	4	−4.44	−3.48	0.95	1.26/983.44	0.307	1.48	4.088	H→L (46.0%)
	5	−4.43	−3.52	0.91	1.21/1027.44	0.300	5.10	5.102	H→L (41.3%)
	∞			0.76					
**P8**	1	−4.76	−3.32	1.45	1.61/768.01	0.17	2.28	0.614	H→L (93.0%)
	2	−4.49	−3.40	1.09	1.33/935.70	0.248	4.37	1.976	H→L (72.4%)
	3	−4.40	−3.44	0.95	1.22/1014.08	0.265	5.70	3.424	H→L (64.5%)
	4	−4.34	−3.48	0.85	1.16/1067.48	0.311	4.03	4.450	H→L (49.6%)
	5	−4.31	−3.50	0.81	1.11/1118.29	0.295	3.91	5.361	H→L (41.3%)
	∞			0.67					
**P16**	1	−5.42	−3.69	1.73	1.83/678.57	0.010	7.13	0.446	H→L (96.1%)
	2	−4.97	−3.91	1.06	1.20/1036.29	0.140	12.52	1.118	H→L (89.5%)
	3	−4.79	−4.01	0.78	0.95/1303.57	0.169	15.63	2.046	H→L (84.5%)
	4	−4.68	−4.06	0.62	0.80/1559.70	0.179	21.60	2.955	H→L (78.5%)
	5	−4.60	−4.10	0.50	0.69/1808.73	0.182	25.73	3.755	H→L (73.7%)
	∞			0.25					
**P18**	1	−5.06	−3.75	1.31	1.48/844.99	0.170	5.83	0.575	H→L (91.7%)
	2	−4.79	−3.84	0.95	1.18/1053.47	0.230	5.88	1.799	H→L (75.0%)
	3	−4.70	−3.89	0.81	1.07/1163.67	0.250	8.07	3.157	H→L (65.2%)
	4	−4.66	−3.91	0.75	1.01/1224.89	0.261	7.53	4.469	H→L (54.7%)
	5	−4.64	−3.92	0.72	0.98/1260.96	0.269	8.66	5.788	H→L (46.2%)
	∞			0.57					
**P19**	1	−5.03	−3.77	1.26	1.40/885.14	0.151	6.18	0.590	H→L (92.1%)
	2	−4.74	−3.86	0.88	1.10/1123.60	0.082	11.19	1.868	H→L (74.4%)
	3	−4.65	−3.92	0.73	0.97/1283.29	0.185	13.04	3.269	H→L (65.4%)
	4	−4.59	−3.92	0.67	0.91/1357.46	0.218	19.16	4.404	H→L (54.4%)
	5	−4.60	−3.97	0.63	0.88/1408.97	0.255	9.47	6.125	H→L (47.3%)
	∞			0.47					

## Data Availability

Data are contained within the article or Supplementary Materials.

## Supplementary Materials

The following supporting information can be downloaded at: https://www.mdpi.com/article/10.3390/molecules29071580/s1, Figure S1: Simulated UV-vis absorption spectra of the reference oligomer; Figures S2–S6: Electron density distribution maps of the reference oligomers (monomer, dimer, trimer, tetramer, and pentamer); Table S1: Computed DFT/TDDFT results of reference molecule; Table S2: Cartesian coordinates of the optimized geometry of the studied oligomers.
