# Supplementary material for: Designing Thiadiazoloquinoxaline-Based Conjugated Polymers for Efficient Organic Photovoltaics: A DFT/TDDFT Study
Source: Molecules. 2024 Apr 1;29(7):1580. doi: 10.3390/molecules29071580 (PMC11013080; doi:10.3390/molecules29071580)
Supplement: Supplementary file 1 [file molecules-29-01580-s001.zip › molecules-2895875-supplementary.pdf]

**Supplementary Material**

# Designing Thiadiazoloquinoxaline-Based Conjugated Polymers for Efficient Organic Photovoltaics: A DFT/TDDFT Study

*Taylor A. Dorlus 1, Juganta K. Roy 1,2 and Jerzy Leszczynski 1,**

*1 Interdisciplinary Center for Nanotoxicity, Jackson State University, Jackson, MS 39217, USA; taylor.dorlus@icnanotox.org (T.A.D.); jroy@wtamu.edu (J.K.R.)*

*2 Clean Energy Materials Modeling Laboratory, Department of Chemistry and Physics, West Texas A&M University, Canyon, TX 79016, USA*

** Correspondence: jerzy@icnanotox.org*

**Table S1**. Computed at the level of theory B3LYP/6-311G(d,p) for ground state and B3LYP/6-311G(d,p)// CAM-B3LYP/6-311G(d,p) for TDDFT calculations. As we found that atactic and syndiotactic oligomers not affecting DFT and TDDFT results significantly (see Figure 1a), here we reported the results of syndiotactic oligomers.

| *Name* | *n* | $E_{HOMO}$ *(eV)* | $E_{LUMO}$ *(eV)* | $E_{gap}$ *(eV)* | $E_{abs}/\lambda_{max}$ *(eV/nm)* | $E_b$ *(eV)* | $\mu_D$ *Debye* | *f* | $S_0 \rightarrow S_1$ *(major contributions)* |
|---|---|---|---|---|---|---|---|---|---|
| | 1 | -5.09 | -3.13 | 1.96 | 2.12/585.95 | 0.16 | 2.92 | 0.452 | H → L (95.8%) |
| | 2 | -4.62 | -3.35 | 1.27 | 1.46/851.69 | 0.19 | 6.87 | 1.267 | H → L (90.1%) |
| **ref** | 3 | -4.44 | -3.44 | 1.00 | 1.24/999.11 | 0.24 | 6.65 | 2.312 | H → L (82.7%) |
| | 4 | -4.35 | -3.49 | 0.86 | 1.12/1110.90 | 0.28 | 7.38 | 3.363 | H → L (75.7%) |
| | 5 | -4.29 | -3.55 | 0.74 | 0.98/1262.60 | 0.24 | 8.58 | 4.449 | H → L (71.4%) |
| | ∞ | | | 0.52 | | | | | |

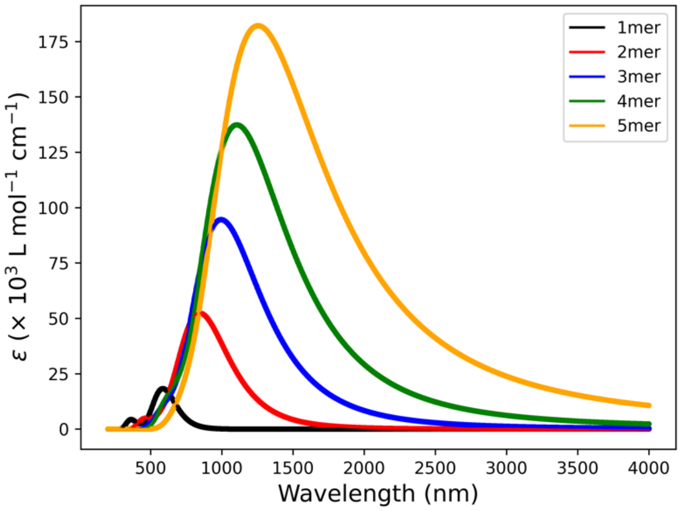


**Figure S1**. Simulated UV-vis absorption spectra of the reference oligomer.

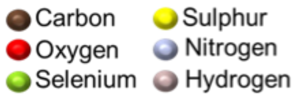


| Repeat units | HOMO | LUMO |
|---|---|---|
| 1 | 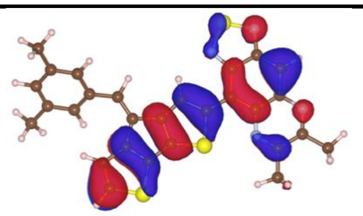 | 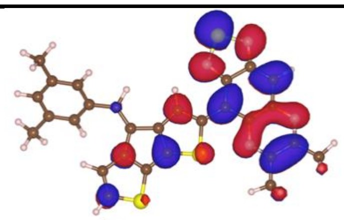 |
| 2 | 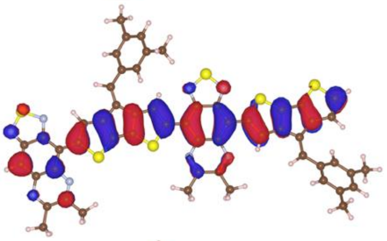 | 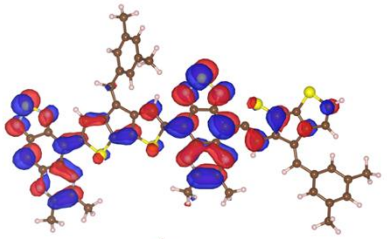 |
| 3 | 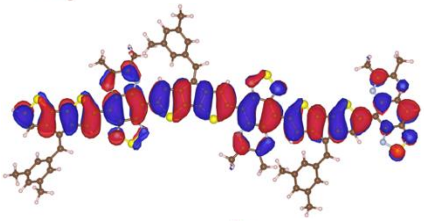 | 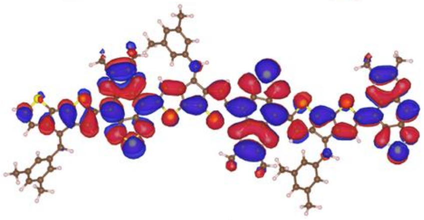 |
| 4 | 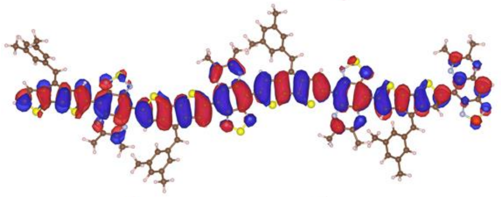 | 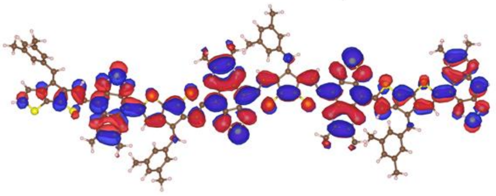 |
| 5 | 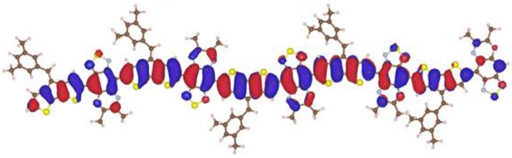 | 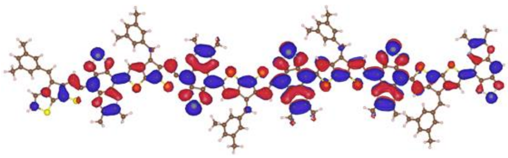 |

**Figure S2**. Electron density distribution of the reference oilgomer with iso value= 0.01 au.

| Name | HOMO | LUMO |
|---|---|---|
| p5 | 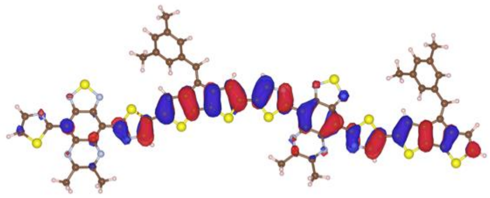 | 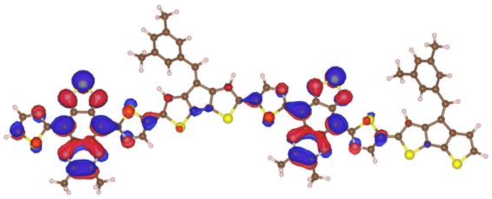 |
| p6 | 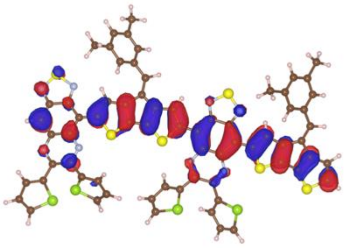 | 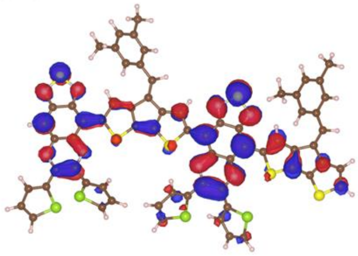 |
| p7 | 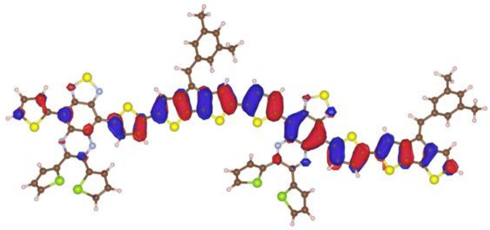 | 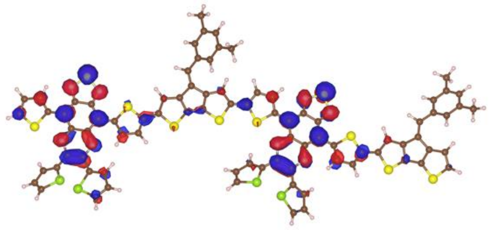 |
| p8 | 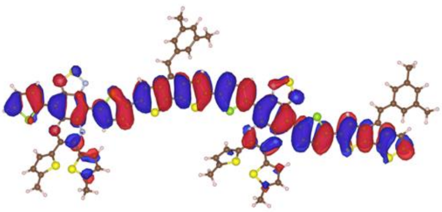 | 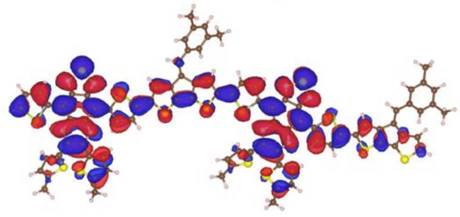 |
| p16 | 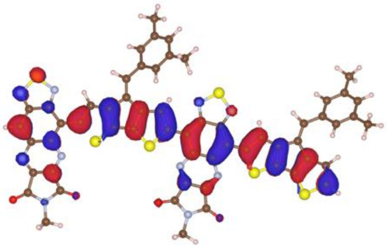 | 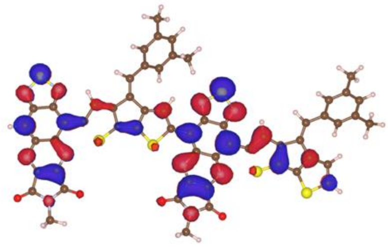 |
| p18 | 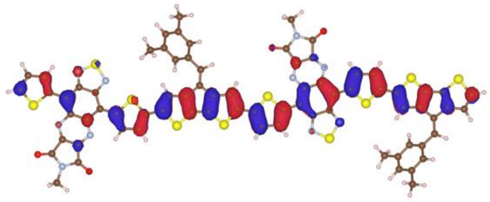 | 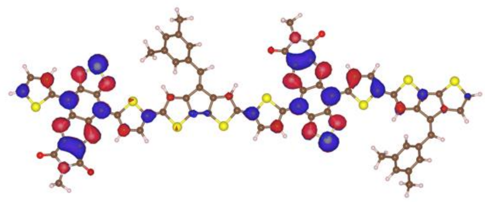 |
| p19 | 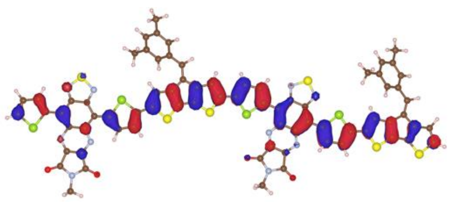 | 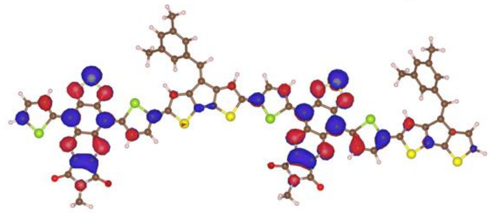 |

**Figure S3**. Electron density distribution of the designed oligomers, in this figure dimers are depicted.

| Name | HOMO | LUMO |
|---|---|---|
| p5 | 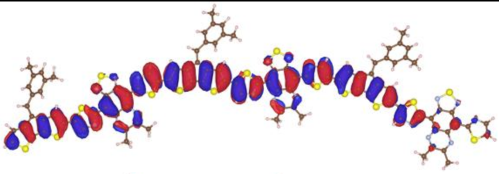 | 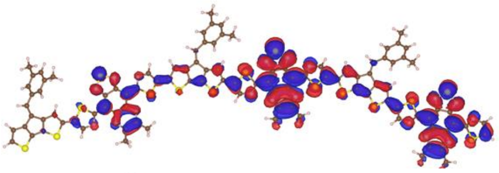 |
| p6 | 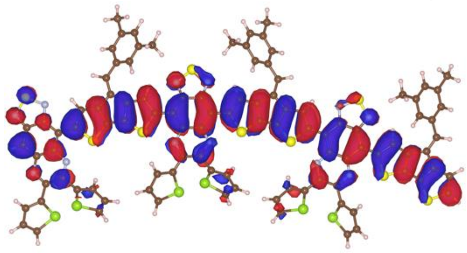 | 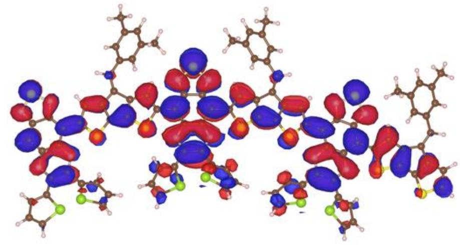 |
| p7 | 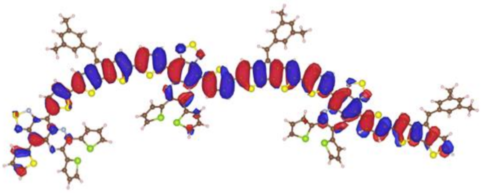 | 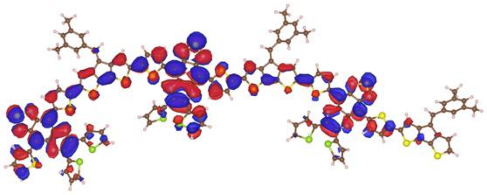 |
| p8 | 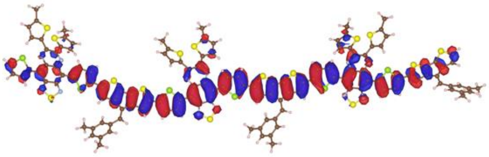 | 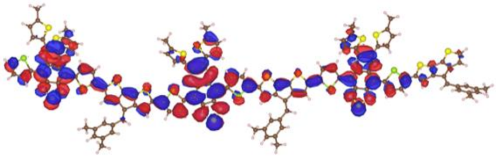 |
| p16 | 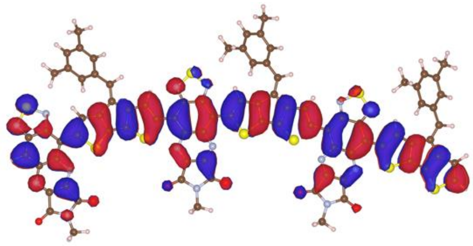 | 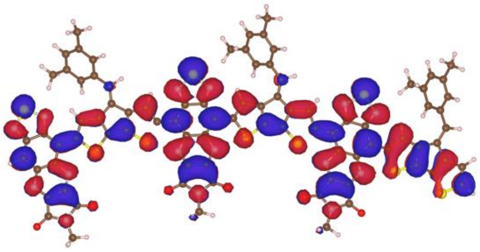 |
| p18 | 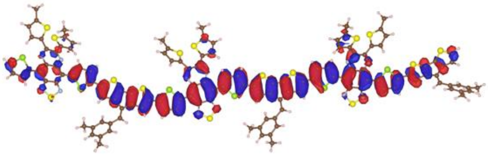 | 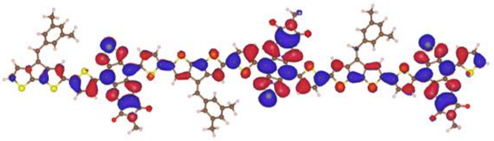 |
| p19 | 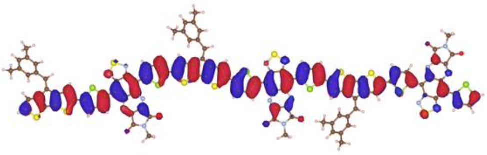 | 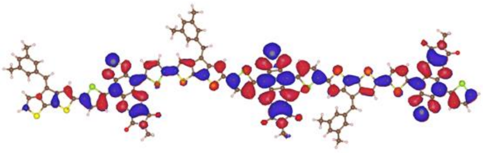 |

**Figure S4**. Electron density distribution of the designed oligomers, in this figure trimers are depicted.

| Name | HOMO | LUMO |
| --- | --- | --- |
| p5 | 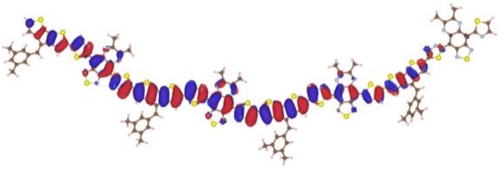 | 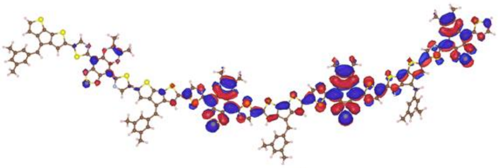 |
| p6 | 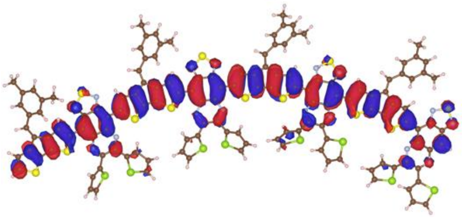 | 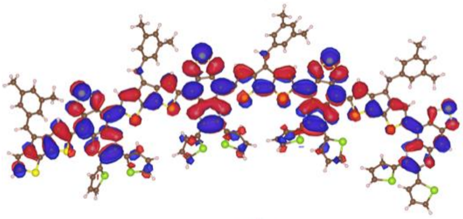 |
| p7 | 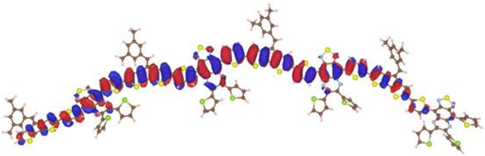 | 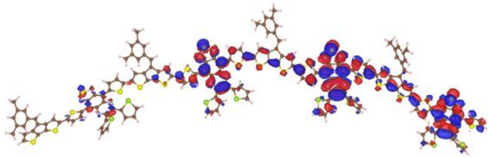 |
| p8 | 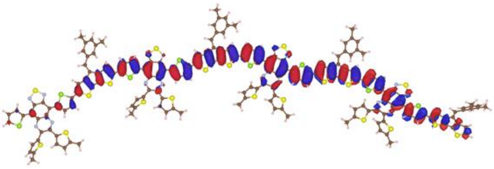 | 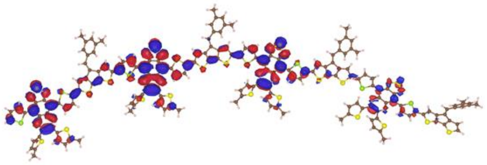 |
| p16 | 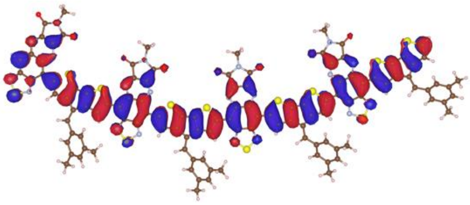 | 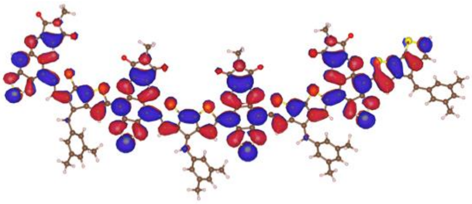 |
| p18 | 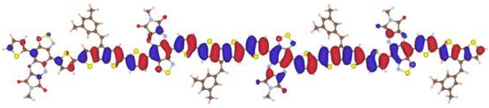 | 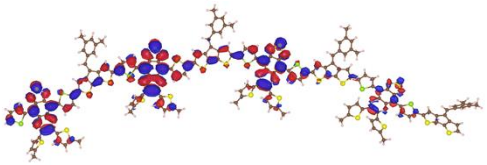 |
| p19 | 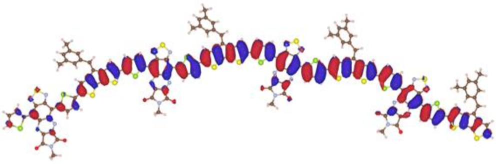 | 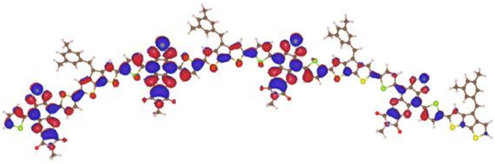 |

**Figure S5**. Electron density distribution of the designed oligomers, in this figure tetramers are depicted.

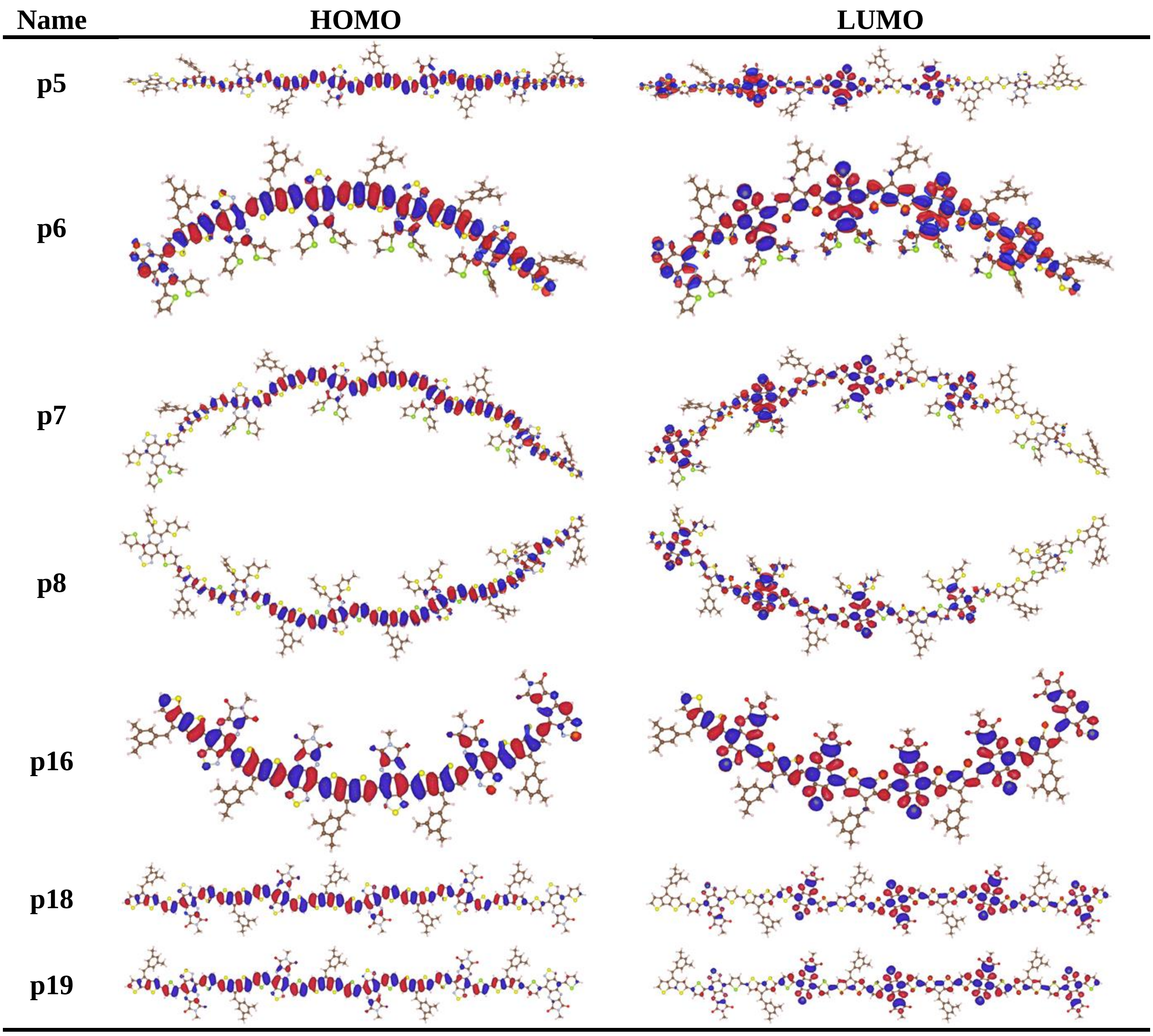


**Figure S6**. Electron density distribution of the designed oligomers, in this figure pentamers are depicted.

Table S2. XYZ coordinates of the ground state geometry of the studied monomers.

| **p05** | | | |
|---|---|---|---|
| Atom | X | Y | Z |
| C | -4.269547 | 1.517282 | -0.20601 |
| C | -2.922069 | 0.950391 | -0.21287 |
| C | -3.819559 | -1.250518 | 0.085385 |
| C | -5.155525 | -0.697239 | 0.032432 |
| C | -5.424072 | 0.676459 | -0.1365 |
| N | -1.961419 | 1.879539 | -0.29618 |
| N | -4.261461 | 2.856377 | -0.2713 |
| S | -2.708942 | 3.329619 | -0.34096 |
| N | -6.225112 | -1.544667 | 0.140597 |
| N | -3.658148 | -2.574557 | 0.386661 |
| C | -6.040491 | -2.816734 | 0.349705 |
| C | -4.699546 | -3.342343 | 0.526869 |
| C | 3.387572 | -0.4089 | -0.08993 |
| C | 4.748668 | -0.787714 | -0.02859 |
| C | 4.914334 | -2.171139 | -0.11801 |
| S | 3.418342 | -3.014823 | -0.2362 |
| C | 6.315911 | -2.480477 | -0.16827 |
| C | 7.032428 | -1.291525 | -0.12096 |
| C | 6.089952 | -0.157329 | -0.01695 |
| C | 6.47792 | 1.137851 | 0.027378 |
| S | 7.34608 | -3.862269 | -0.25412 |
| C | 8.757141 | -2.827602 | -0.22579 |
| C | 8.436868 | -1.499452 | -0.14894 |
| C | 5.328909 | 4.708412 | -0.2832 |
| C | 6.041918 | 3.523398 | -0.4494 |
| C | 5.680991 | 2.350246 | 0.233474 |
| C | 4.611577 | 2.40228 | 1.13819 |
| C | 3.894031 | 3.582286 | 1.342106 |
| C | 4.25698 | 4.719305 | 0.615966 |
| C | 5.687414 | 5.948652 | -1.06641 |
| H | 6.708798 | 5.897258 | -1.44904 |
| H | 5.019494 | 6.07538 | -1.92508 |
| H | 5.600222 | 6.84752 | -0.45089 |
| C | 2.777126 | 3.638153 | 2.357545 |
| H | 2.251815 | 2.68318 | 2.428323 |
| H | 3.166861 | 3.872065 | 3.354179 |
| H | 2.046428 | 4.408608 | 2.102222 |
| S | 0.127797 | -0.088708 | 0.137035 |
| C | 1.088123 | -1.483565 | -0.27498 |
| C | -1.34096 | -1.008053 | -0.26347 |
| C | 0.240626 | -2.493947 | -0.69333 |
| C | 2.525757 | -1.483101 | -0.20107 |

| | | | |
|---|---|---|---|
| C | -2.675967 | -0.44955 | -0.12746 |
| C | -6.772669 | 1.225308 | -0.24577 |
| C | -9.174747 | 1.722219 | -0.28563 |
| C | -8.339583 | 2.766003 | -0.56439 |
| S | -8.264161 | 0.286747 | 0.012454 |
| H | -8.656002 | 3.773521 | -0.80031 |
| H | -10.25369 | 1.715994 | -0.257 |
| H | 9.185667 | -0.718982 | -0.11742 |
| H | 9.736151 | -3.278667 | -0.27046 |
| H | 3.030794 | 0.610789 | -0.07962 |
| H | 0.577945 | -3.459605 | -1.04839 |
| H | 4.36422 | 1.520424 | 1.717282 |
| H | 6.883239 | 3.497857 | -1.13518 |
| H | 3.700961 | 5.640657 | 0.764615 |
| H | 7.540709 | 1.319313 | -0.11955 |
| N | -1.082404 | -2.225727 | -0.67976 |
| C | -4.469245 | -4.780771 | 0.901219 |
| H | -4.94482 | -5.027041 | 1.856379 |
| H | -4.888029 | -5.458742 | 0.150598 |
| H | -3.398087 | -4.957512 | 0.979913 |
| C | -7.245809 | -3.711115 | 0.428883 |
| H | -7.200867 | -4.496955 | -0.33195 |
| H | -7.304098 | -4.211754 | 1.400512 |
| H | -8.149757 | -3.123816 | 0.277761 |
| N | -7.010105 | 2.480238 | -0.53792 |
| **p06** | | | |
| C | 1.011304 | 4.368437 | -0.33243 |
| C | -0.050543 | 3.368063 | -0.21675 |
| C | 0.248266 | 1.962656 | -0.09533 |
| C | 1.635728 | 1.657513 | -0.09365 |
| C | 2.671158 | 2.666819 | -0.18472 |
| C | 2.36672 | 4.012145 | -0.31253 |
| N | -1.270576 | 3.920034 | -0.24815 |
| N | 0.548492 | 5.625813 | -0.44403 |
| S | -1.07773 | 5.53388 | -0.40655 |
| N | 3.986526 | 2.299306 | -0.10117 |
| N | 2.008901 | 0.352174 | -0.04939 |
| C | 4.311937 | 1.033455 | 0.001825 |
| C | 3.271127 | 0.00822 | -0.0379 |
| C | -0.788984 | 0.96031 | 0.002585 |
| C | -2.167265 | 1.181072 | 0.020081 |
| C | -2.920924 | 0.007502 | 0.139998 |
| C | -2.126505 | -1.134862 | 0.208704 |
| S | -0.441989 | -0.792727 | 0.124978 |
| C | -2.990726 | -2.273541 | 0.318448 |
| C | -4.323665 | -1.853785 | 0.311112 |

| | | | |
|---|---|---|---|
| C | -4.348964 | -0.379529 | 0.18188 |
| C | -5.368251 | 0.508844 | 0.140248 |
| S | -2.835575 | -3.975106 | 0.557365 |
| C | -4.573037 | -4.130657 | 0.667824 |
| C | -5.22284 | -2.934948 | 0.526091 |
| C | -8.749179 | -0.924934 | -0.80516 |
| C | -7.368999 | -0.753512 | -0.71311 |
| C | -6.813209 | 0.278465 | 0.059405 |
| C | -7.68061 | 1.162525 | 0.718007 |
| C | -9.065161 | 1.001453 | 0.661717 |
| C | -9.582253 | -0.047607 | -0.10236 |
| C | -9.338529 | -2.009066 | -1.67666 |
| C | -9.981684 | 1.927357 | 1.425609 |
| H | -10.16519 | -2.518341 | -1.17403 |
| H | -9.73291 | -1.591563 | -2.60909 |
| H | -8.590055 | -2.758232 | -1.94204 |
| H | -7.261549 | 1.980127 | 1.296725 |
| H | -6.712425 | -1.401285 | -1.28128 |
| H | -6.297575 | -2.846182 | 0.583268 |
| H | -5.002712 | -5.107468 | 0.827238 |
| H | -2.574301 | 2.176717 | -0.04937 |
| H | 3.150713 | 4.753072 | -0.37479 |
| H | -10.65933 | -0.174863 | -0.16636 |
| H | -9.546802 | 2.924366 | 1.526168 |
| H | -10.95023 | 2.027842 | 0.930572 |
| H | -10.16765 | 1.548078 | 2.436282 |
| H | -5.08198 | 1.557893 | 0.176203 |
| C | 5.742156 | 0.75501 | 0.188279 |
| C | 6.751131 | 1.62168 | -0.15343 |
| C | 8.133735 | 0.063087 | 0.925776 |
| C | 8.062039 | 1.237672 | 0.235564 |
| H | 6.530149 | 2.544234 | -0.67351 |
| H | 9.016758 | -0.420534 | 1.316074 |
| H | 8.937102 | 1.832999 | 0.004919 |
| C | 3.551174 | -1.440903 | -0.08877 |
| C | 3.157539 | -2.386601 | 0.815769 |
| C | 4.074454 | -3.900839 | -0.73195 |
| C | 3.459501 | -3.738822 | 0.471391 |
| H | 2.651119 | -2.112534 | 1.733168 |
| H | 4.389498 | -4.82346 | -1.19548 |
| H | 3.214369 | -4.573138 | 1.117222 |
| Se | 4.359702 | -2.264631 | -1.59551 |
| Se | 6.455135 | -0.732188 | 1.129366 |
| **p07** | | | |
| C | -1.78953 | 3.3252 | 0.088399 |
| C | -0.685785 | 2.370174 | -0.01716 |

| | | | |
|---|---|---|---|
| C | -2.231609 | 0.550896 | 0.034987 |
| C | -3.326754 | 1.504252 | 0.028942 |
| C | -3.160476 | 2.907046 | 0.069361 |
| N | 0.514765 | 2.960467 | -0.01454 |
| N | -1.357273 | 4.591099 | 0.166187 |
| S | 0.271441 | 4.567444 | 0.11287 |
| N | -4.592576 | 1.013679 | -0.08109 |
| N | -2.519105 | -0.757855 | 0.239312 |
| C | -4.838688 | -0.267502 | -0.02633 |
| C | -3.759276 | -1.191772 | 0.294875 |
| C | 4.942737 | -0.59672 | -0.20383 |
| C | 6.1855 | -1.266054 | -0.14214 |
| C | 6.042837 | -2.652042 | -0.24011 |
| S | 4.397131 | -3.144229 | -0.3632 |
| C | 7.342275 | -3.260858 | -0.2891 |
| C | 8.302796 | -2.258783 | -0.22938 |
| C | 7.632419 | -0.945775 | -0.11972 |
| C | 8.296598 | 0.231459 | -0.05851 |
| S | 8.044667 | -4.834543 | -0.3843 |
| C | 9.648709 | -4.135341 | -0.34299 |
| C | 9.627231 | -2.769923 | -0.25532 |
| C | 8.000906 | 3.972186 | -0.33319 |
| C | 8.427132 | 2.658238 | -0.50342 |
| C | 7.787713 | 1.589797 | 0.148967 |
| C | 6.732983 | 1.875121 | 1.025604 |
| C | 6.292042 | 3.185644 | 1.225643 |
| C | 6.929977 | 4.217561 | 0.53484 |
| C | 8.666435 | 5.109881 | -1.07016 |
| H | 9.629559 | 4.80672 | -1.48545 |
| H | 8.042766 | 5.457386 | -1.90055 |
| H | 8.835594 | 5.965533 | -0.41108 |
| C | 5.144611 | 3.471329 | 2.16557 |
| H | 4.185633 | 3.207603 | 1.70718 |
| H | 5.232078 | 2.890835 | 3.087535 |
| H | 5.103903 | 4.528852 | 2.434086 |
| S | 1.894275 | 0.45794 | 0.077637 |
| C | 2.46272 | -1.132079 | -0.3753 |
| C | 0.216686 | 0.033503 | -0.27525 |
| C | 1.406619 | -1.925097 | -0.78884 |
| C | 0.163012 | -1.277756 | -0.73225 |
| C | 3.863297 | -1.453526 | -0.31753 |
| C | -0.877586 | 0.958029 | -0.09537 |
| C | -4.263605 | 3.855779 | 0.072275 |
| C | -6.4757 | 5.034422 | -0.00806 |
| C | -4.181552 | 5.235389 | 0.195219 |
| C | -5.428988 | 5.898701 | 0.150976 |

| | | | |
|---|---|---|---|
| S | -5.961958 | 3.388108 | -0.10321 |
| H | -3.239414 | 5.743698 | 0.313899 |
| H | -5.539294 | 6.971994 | 0.233398 |
| H | -7.527985 | 5.267728 | -0.0784 |
| H | 10.528865 | -2.173001 | -0.21471 |
| H | 10.504978 | -4.789965 | -0.38898 |
| H | 4.820403 | 0.476399 | -0.19391 |
| H | 1.5356 | -2.935767 | -1.15409 |
| H | -0.762285 | -1.737679 | -1.03794 |
| H | 6.278931 | 1.068536 | 1.588714 |
| H | 9.263797 | 2.448906 | -1.16282 |
| H | 6.598345 | 5.240517 | 0.688632 |
| H | 9.374629 | 0.173282 | -0.19461 |
| C | -6.223674 | -0.684239 | -0.31912 |
| C | -7.362991 | -0.272 | 0.31337 |
| C | -8.451097 | -1.507932 | -1.36531 |
| C | -8.587901 | -0.738777 | -0.25032 |
| H | -7.32147 | 0.366929 | 1.186647 |
| H | -9.231969 | -1.96133 | -1.95691 |
| H | -9.554443 | -0.49631 | 0.174222 |
| C | -3.942663 | -2.56728 | 0.765703 |
| C | -2.915822 | -3.456538 | 0.988044 |
| C | -4.609034 | -4.838976 | 1.83944 |
| C | -3.279971 | -4.702154 | 1.560874 |
| H | -1.894082 | -3.18775 | 0.757292 |
| H | -5.108088 | -5.69265 | 2.273278 |
| H | -2.55685 | -5.481599 | 1.7681 |
| Se | -6.655009 | -1.742766 | -1.83521 |
| Se | -5.585697 | -3.323911 | 1.349743 |
| **p08** | | | |
| C | -2.175324 | 3.114629 | -0.19102 |
| C | -0.995658 | 2.253132 | -0.23893 |
| C | -2.370714 | 0.313357 | -0.02462 |
| C | -3.545198 | 1.165552 | -0.08315 |
| C | -3.504661 | 2.577667 | -0.1633 |
| N | 0.149104 | 2.941544 | -0.28564 |
| N | -1.853547 | 4.414989 | -0.20639 |
| S | -0.228587 | 4.52972 | -0.26933 |
| N | -4.765112 | 0.560452 | -0.12408 |
| N | -2.538606 | -0.994092 | 0.287562 |
| C | -4.897727 | -0.728837 | 0.041822 |
| C | -3.736159 | -1.525301 | 0.407562 |
| C | 5.009686 | -0.529879 | -0.21478 |
| C | 6.261972 | -1.176825 | -0.29676 |
| C | 6.138725 | -2.517342 | -0.67118 |
| S | 4.501736 | -2.998143 | -0.89788 |

| | | | |
|---|---|---|---|
| C | 7.44568 | -3.084899 | -0.84123 |
| C | 8.392864 | -2.101519 | -0.58264 |
| C | 7.70473 | -0.847861 | -0.20845 |
| C | 8.353393 | 0.300969 | 0.090971 |
| S | 8.169146 | -4.597442 | -1.25161 |
| C | 9.763557 | -3.897908 | -1.07256 |
| C | 9.723805 | -2.578152 | -0.71252 |
| C | 7.988732 | 4.012953 | 0.595842 |
| C | 8.436609 | 2.769832 | 0.153585 |
| C | 7.827962 | 1.578424 | 0.580045 |
| C | 6.783649 | 1.655221 | 1.512704 |
| C | 6.329872 | 2.885449 | 1.990398 |
| C | 6.933389 | 4.052105 | 1.512884 |
| C | 8.611609 | 5.289963 | 0.084263 |
| H | 9.618192 | 5.115782 | -0.3017 |
| H | 8.016238 | 5.716941 | -0.72992 |
| H | 8.675801 | 6.045222 | 0.871437 |
| C | 5.239813 | 2.952362 | 3.033949 |
| H | 4.563684 | 2.097864 | 2.96257 |
| H | 5.665313 | 2.950843 | 4.043347 |
| H | 4.646168 | 3.86354 | 2.930989 |
| C | 2.536205 | -1.057004 | -0.48497 |
| C | 0.119597 | 0.010086 | -0.35705 |
| C | 1.47633 | -1.915299 | -0.70507 |
| C | 0.193049 | -1.345754 | -0.63855 |
| C | 3.937689 | -1.360059 | -0.49613 |
| C | -1.057479 | 0.826869 | -0.21641 |
| C | -4.687947 | 3.41383 | -0.23567 |
| C | -7.056705 | 4.497747 | -0.44956 |
| C | -4.740006 | 4.795574 | -0.22507 |
| C | -6.026465 | 5.383655 | -0.33922 |
| Se | 1.89646 | 0.708657 | -0.19707 |
| Se | -6.465355 | 2.720184 | -0.40616 |
| H | 1.620475 | -2.967738 | -0.92148 |
| H | -0.701309 | -1.925389 | -0.80333 |
| H | -3.843179 | 5.387829 | -0.13515 |
| H | -6.168299 | 6.458071 | -0.33899 |
| H | -8.107447 | 4.729539 | -0.54974 |
| H | 4.87463 | 0.511705 | 0.038514 |
| H | 10.617539 | -1.989091 | -0.55407 |
| H | 10.628599 | -4.517651 | -1.24997 |
| H | 9.261531 | 2.718744 | -0.55051 |
| H | 6.349257 | 0.740661 | 1.89837 |
| H | 6.583423 | 5.014551 | 1.875475 |
| H | 9.43135 | 0.28781 | -0.05712 |
| C | -3.791543 | -2.869512 | 0.985292 |

| | | | |
|---|---|---|---|
| C | -2.697802 | -3.671633 | 1.230925 |
| S | -5.238798 | -3.623818 | 1.637255 |
| C | -3.011387 | -4.873248 | 1.906397 |
| H | -1.699197 | -3.372826 | 0.947628 |
| C | -4.342921 | -5.000844 | 2.209515 |
| H | -2.276947 | -5.623885 | 2.170186 |
| C | -6.244058 | -1.2922 | -0.17307 |
| C | -7.414046 | -0.933909 | 0.440751 |
| S | -6.552314 | -2.464914 | -1.43813 |
| C | -8.551304 | -1.616782 | -0.07177 |
| H | -7.448651 | -0.207768 | 1.241573 |
| C | -8.262028 | -2.477835 | -1.09499 |
| H | -9.556842 | -1.473386 | 0.303336 |
| C | -5.029275 | -6.128662 | 2.917144 |
| H | -5.537194 | -5.789579 | 3.824912 |
| H | -5.778233 | -6.611098 | 2.281804 |
| H | -4.296074 | -6.88438 | 3.205169 |
| C | -9.20249 | -3.342295 | -1.87835 |
| H | -9.195623 | -3.088609 | -2.94252 |
| H | -8.950086 | -4.40305 | -1.79017 |
| H | -10.22063 | -3.20688 | -1.50841 |
| **p16** | | | |
| C | -2.943265 | 3.65388 | 0.123329 |
| C | -1.771268 | 2.782665 | 0.073732 |
| C | -1.897897 | 1.343879 | 0.029785 |
| C | -3.242752 | 0.870358 | 0.038245 |
| C | -4.400639 | 1.7688 | 0.087802 |
| C | -4.249743 | 3.140511 | 0.130013 |
| N | -0.626835 | 3.476594 | 0.07482 |
| N | -2.635787 | 4.960604 | 0.160071 |
| S | -1.010128 | 5.062619 | 0.133493 |
| N | -5.692361 | 1.267442 | 0.09409 |
| N | -3.43277 | -0.477279 | -0.00181 |
| C | -5.78218 | -0.022169 | 0.053568 |
| C | -4.661388 | -0.897508 | 0.005751 |
| C | -5.155555 | -2.307001 | -0.03103 |
| C | -7.028447 | -0.865782 | 0.04806 |
| N | -6.560635 | -2.189749 | -0.00282 |
| O | -8.186624 | -0.540564 | 0.078842 |
| O | -4.541838 | -3.342952 | -0.07499 |
| C | -7.45496 | -3.335956 | -0.02431 |
| C | -0.751929 | 0.472058 | -0.01756 |
| C | 0.593459 | 0.861551 | -0.02138 |
| C | 1.485324 | -0.209611 | -0.08174 |
| C | 0.836047 | -1.446325 | -0.11535 |
| S | -0.878015 | -1.315658 | -0.08002 |

| | | | |
|---|---|---|---|
| C | 1.832534 | -2.470968 | -0.16817 |
| C | 3.105412 | -1.888797 | -0.1579 |
| C | 2.949963 | -0.419586 | -0.09095 |
| C | 3.85266 | 0.588107 | -0.08358 |
| S | 1.890764 | -4.187427 | -0.33384 |
| C | 3.634137 | -4.130006 | -0.40769 |
| C | 4.13315 | -2.859035 | -0.30864 |
| C | 7.373514 | -0.369821 | 0.946097 |
| C | 5.983748 | -0.374538 | 0.841277 |
| C | 5.313599 | 0.540954 | 0.014367 |
| C | 6.071252 | 1.49012 | -0.68736 |
| C | 7.464647 | 1.502984 | -0.61957 |
| C | 8.098605 | 0.566193 | 0.20022 |
| C | 8.08613 | -1.329017 | 1.870297 |
| C | 8.268584 | 2.493075 | -1.42812 |
| H | -8.102682 | -3.293493 | -0.90191 |
| H | -8.079983 | -3.343728 | 0.870489 |
| H | -6.839663 | -4.233128 | -0.05793 |
| H | 8.92934 | -1.811526 | 1.368311 |
| H | 8.485671 | -0.80695 | 2.745809 |
| H | 7.414081 | -2.110539 | 2.229746 |
| H | 5.560174 | 2.21921 | -1.30873 |
| H | 5.405967 | -1.067448 | 1.44098 |
| H | 5.19021 | -2.643162 | -0.3515 |
| H | 4.183703 | -5.052083 | -0.51833 |
| H | 0.874503 | 1.901252 | 0.017398 |
| H | -5.115179 | 3.786363 | 0.166447 |
| H | 9.182497 | 0.576074 | 0.27431 |
| H | 7.710475 | 3.416837 | -1.5962 |
| H | 9.205654 | 2.748511 | -0.92833 |
| H | 8.522757 | 2.080738 | -2.41056 |
| H | 3.440523 | 1.59127 | -0.17076 |
| **p18** | | | |
| C | -3.40641 | 2.192734 | -0.02131 |
| C | -2.187157 | 1.385784 | -0.01007 |
| C | -3.478145 | -0.625745 | -0.02738 |
| C | -4.70261 | 0.191222 | -0.03693 |
| C | -4.71414 | 1.606195 | -0.03639 |
| N | -1.078007 | 2.129503 | 0.00565 |
| N | -3.143129 | 3.506436 | -0.01455 |
| S | -1.525839 | 3.695139 | 0.003934 |
| N | -5.92739 | -0.421806 | -0.04772 |
| N | -3.579751 | -1.987364 | -0.02893 |
| C | -5.948251 | -1.716042 | -0.04901 |
| C | -4.771666 | -2.498456 | -0.03952 |
| C | -5.15863 | -3.943874 | -0.04339 |

| | | | |
|---|---|---|---|
| C | -7.130649 | -2.640195 | -0.05912 |
| N | -6.567801 | -3.929898 | -0.05502 |
| O | -8.310103 | -2.398884 | -0.06871 |
| O | -4.466228 | -4.930102 | -0.03844 |
| C | -7.376891 | -5.138071 | -0.06261 |
| C | 3.830083 | -0.534242 | -0.01304 |
| C | 5.175191 | -0.952815 | 0.031998 |
| C | 5.292658 | -2.347068 | 0.05637 |
| S | 3.768698 | -3.145188 | 0.05851 |
| C | 6.678863 | -2.706841 | -0.00738 |
| C | 7.435742 | -1.542532 | -0.08064 |
| C | 6.536111 | -0.371034 | -0.04464 |
| C | 6.967296 | 0.909541 | -0.11434 |
| S | 7.660502 | -4.126707 | -0.00915 |
| C | 9.103432 | -3.14263 | -0.10905 |
| C | 8.830242 | -1.801675 | -0.13368 |
| C | 5.941819 | 4.486427 | -0.67328 |
| C | 6.606285 | 3.266617 | -0.76334 |
| C | 6.216221 | 2.161125 | 0.012113 |
| C | 5.168091 | 2.320693 | 0.927795 |
| C | 4.492419 | 3.537336 | 1.04972 |
| C | 4.885744 | 4.6035 | 0.238712 |
| H | -8.003859 | -5.167508 | -0.95562 |
| H | -8.021507 | -5.166594 | 0.817756 |
| H | -6.698034 | -5.988853 | -0.05551 |
| C | 6.339099 | 5.65695 | -1.5405 |
| H | 7.32343 | 5.505873 | -1.98789 |
| H | 5.623297 | 5.800654 | -2.3567 |
| H | 6.367004 | 6.586347 | -0.96563 |
| C | 3.357794 | 3.686278 | 2.035802 |
| H | 2.442874 | 3.218898 | 1.656229 |
| H | 3.593935 | 3.205948 | 2.988655 |
| H | 3.136384 | 4.7372 | 2.232313 |
| S | 0.649068 | -0.0026 | 0.007413 |
| C | 1.493015 | -1.532827 | -0.00282 |
| C | -0.939934 | -0.798939 | -0.01274 |
| C | 0.590567 | -2.585774 | -0.01942 |
| C | -0.746579 | -2.182493 | -0.02446 |
| C | 2.925511 | -1.583998 | 0.004613 |
| C | -2.174952 | -0.048549 | -0.01656 |
| C | -5.928857 | 2.407475 | -0.04963 |
| C | -8.269348 | 3.303024 | -0.07358 |
| C | -6.012235 | 3.794741 | -0.0543 |
| C | -7.331179 | 4.298148 | -0.06784 |
| S | -7.564988 | 1.72839 | -0.06298 |
| H | -5.137067 | 4.421779 | -0.04804 |

| | | | |
|---|---|---|---|
| H | -7.569236 | 5.353576 | -0.07317 |
| H | -9.344946 | 3.401693 | -0.08377 |
| H | 9.60521 | -1.048703 | -0.18925 |
| H | 10.065502 | -3.629622 | -0.1445 |
| H | 3.508839 | 0.495594 | -0.06645 |
| H | 0.891078 | -3.625475 | -0.03034 |
| H | -1.574201 | -2.87015 | -0.03754 |
| H | 4.906006 | 1.497962 | 1.582383 |
| H | 7.436246 | 3.159804 | -1.45497 |
| H | 4.37103 | 5.555632 | 0.331166 |
| H | 8.030861 | 1.041263 | -0.30264 |
| **p19** | | | |
| C | -3.31978 | 2.092269 | -0.13461 |
| C | -2.054615 | 1.363132 | -0.11231 |
| C | -3.213571 | -0.725744 | -0.02213 |
| C | -4.486481 | 0.01325 | -0.04365 |
| C | -4.58896 | 1.424853 | -0.10032 |
| N | -0.993358 | 2.171582 | -0.15037 |
| N | -3.138109 | 3.418382 | -0.18899 |
| S | -1.535708 | 3.708424 | -0.21 |
| N | -5.669155 | -0.676171 | -0.0079 |
| N | -3.228034 | -2.088935 | 0.033352 |
| C | -5.608756 | -1.968897 | 0.044063 |
| C | -4.385727 | -2.674609 | 0.064652 |
| C | -4.681619 | -4.13983 | 0.126157 |
| C | -6.731015 | -2.963279 | 0.092101 |
| N | -6.089889 | -4.213774 | 0.13865 |
| O | -7.923146 | -2.79248 | 0.093299 |
| O | -3.929882 | -5.080782 | 0.159232 |
| C | -6.821629 | -5.469103 | 0.194586 |
| C | 4.254615 | -0.470811 | -0.00751 |
| C | 5.588431 | -0.922505 | 0.009549 |
| C | 5.67231 | -2.319713 | -0.0277 |
| S | 4.130736 | -3.081138 | -0.05229 |
| C | 7.048616 | -2.708679 | -0.11685 |
| C | 7.8332 | -1.560531 | -0.14481 |
| C | 6.962635 | -0.370686 | -0.04986 |
| C | 7.424419 | 0.900821 | -0.06386 |
| S | 7.995566 | -4.150277 | -0.18799 |
| C | 9.460975 | -3.197439 | -0.25559 |
| C | 9.220463 | -1.850384 | -0.21957 |
| C | 6.476813 | 4.522093 | -0.44745 |
| C | 7.109413 | 3.292664 | -0.60341 |
| C | 6.705462 | 2.162939 | 0.129754 |
| C | 5.677814 | 2.305703 | 1.070164 |
| C | 5.03733 | 3.533254 | 1.261622 |

| | | | |
|---|---|---|---|
| C | 5.44096 | 4.623799 | 0.490186 |
| H | -7.460461 | -5.573712 | -0.68425 |
| H | -7.448039 | -5.502007 | 1.087858 |
| H | -6.090572 | -6.275041 | 0.222092 |
| C | 6.884518 | 5.719965 | -1.27129 |
| H | 7.850938 | 5.561781 | -1.75384 |
| H | 6.150668 | 5.92231 | -2.0585 |
| H | 6.956463 | 6.620277 | -0.65535 |
| C | 3.944615 | 3.669833 | 2.295799 |
| H | 3.102372 | 3.00718 | 2.074736 |
| H | 4.309992 | 3.405304 | 3.292528 |
| H | 3.564025 | 4.691947 | 2.33925 |
| C | 1.894162 | -1.411172 | -0.02758 |
| C | -0.666012 | -0.722428 | -0.04054 |
| C | 0.978005 | -2.448541 | 0.010345 |
| C | -0.373677 | -2.084062 | 0.004143 |
| C | 3.321201 | -1.497048 | -0.02973 |
| C | -1.948653 | -0.066335 | -0.05681 |
| C | -5.852938 | 2.137194 | -0.12189 |
| C | -8.321564 | 2.990674 | -0.14096 |
| C | -6.035307 | 3.509234 | -0.17818 |
| C | -7.374858 | 3.971833 | -0.18854 |
| Se | 0.98997 | 0.256593 | -0.086 |
| Se | -7.566946 | 1.279159 | -0.07457 |
| H | 1.280956 | -3.488522 | 0.048526 |
| H | -1.163644 | -2.816328 | 0.034453 |
| H | -5.196434 | 4.185553 | -0.21233 |
| H | -7.617733 | 5.02698 | -0.23127 |
| H | -9.394319 | 3.121599 | -0.13928 |
| H | 3.961158 | 0.568961 | -0.00746 |
| H | 10.013169 | -1.114579 | -0.24804 |
| H | 10.410773 | -3.705211 | -0.3195 |
| H | 7.922865 | 3.196363 | -1.31586 |
| H | 5.404606 | 1.459473 | 1.689672 |
| H | 4.950607 | 5.582403 | 0.633313 |
| H | 8.489559 | 1.015767 | -0.25433 |
